# Supplementary figures and images for: Requirement of Nek2a and cyclin A2 for Wapl-dependent removal of cohesin from prophase chromatin
Source: EMBO J. 2024 Sep 13;43(21):20. doi: 10.1038/s44318-024-00228-9 (PMC11535040; doi:10.1038/s44318-024-00228-9)

Fig.1B

supernatant after centrifugation

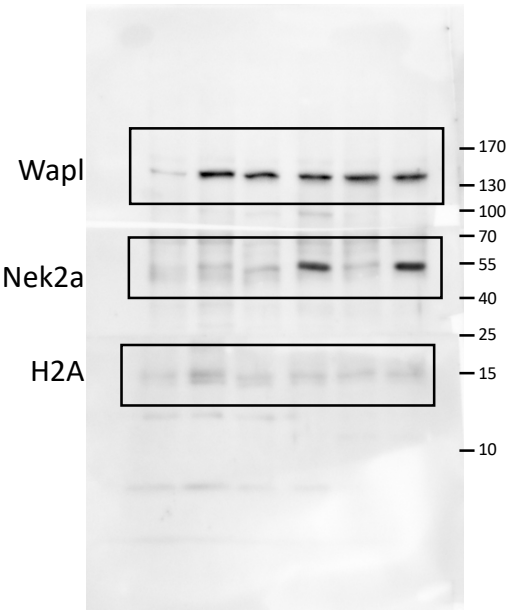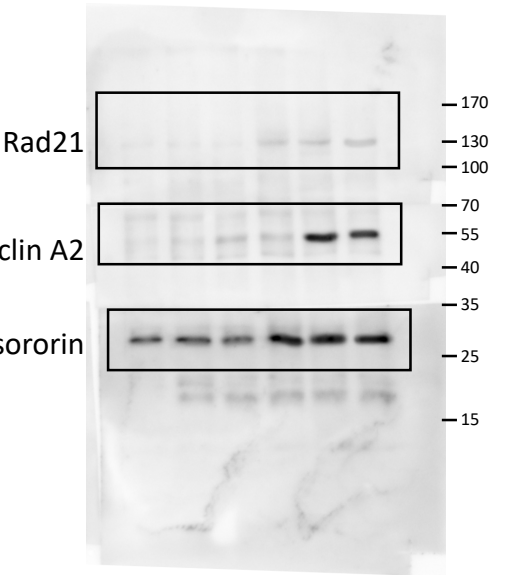

pelleted chromatin

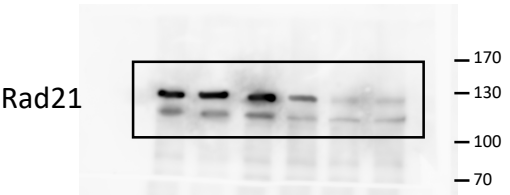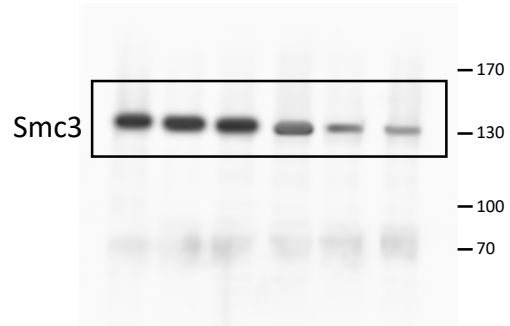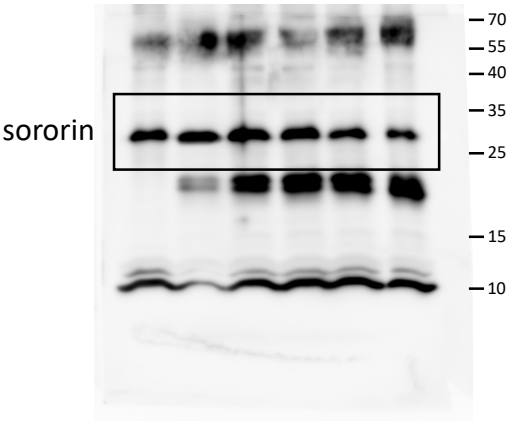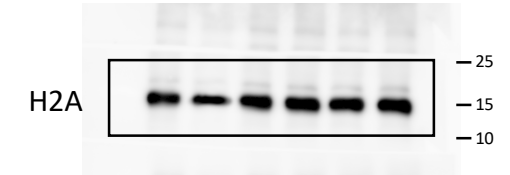

Supplement: Supplementary file 2 — Source data Fig. 1 [file 44318_2024_228_MOESM2_ESM.zip › Figure1/1B.pdf]

Fig.1C

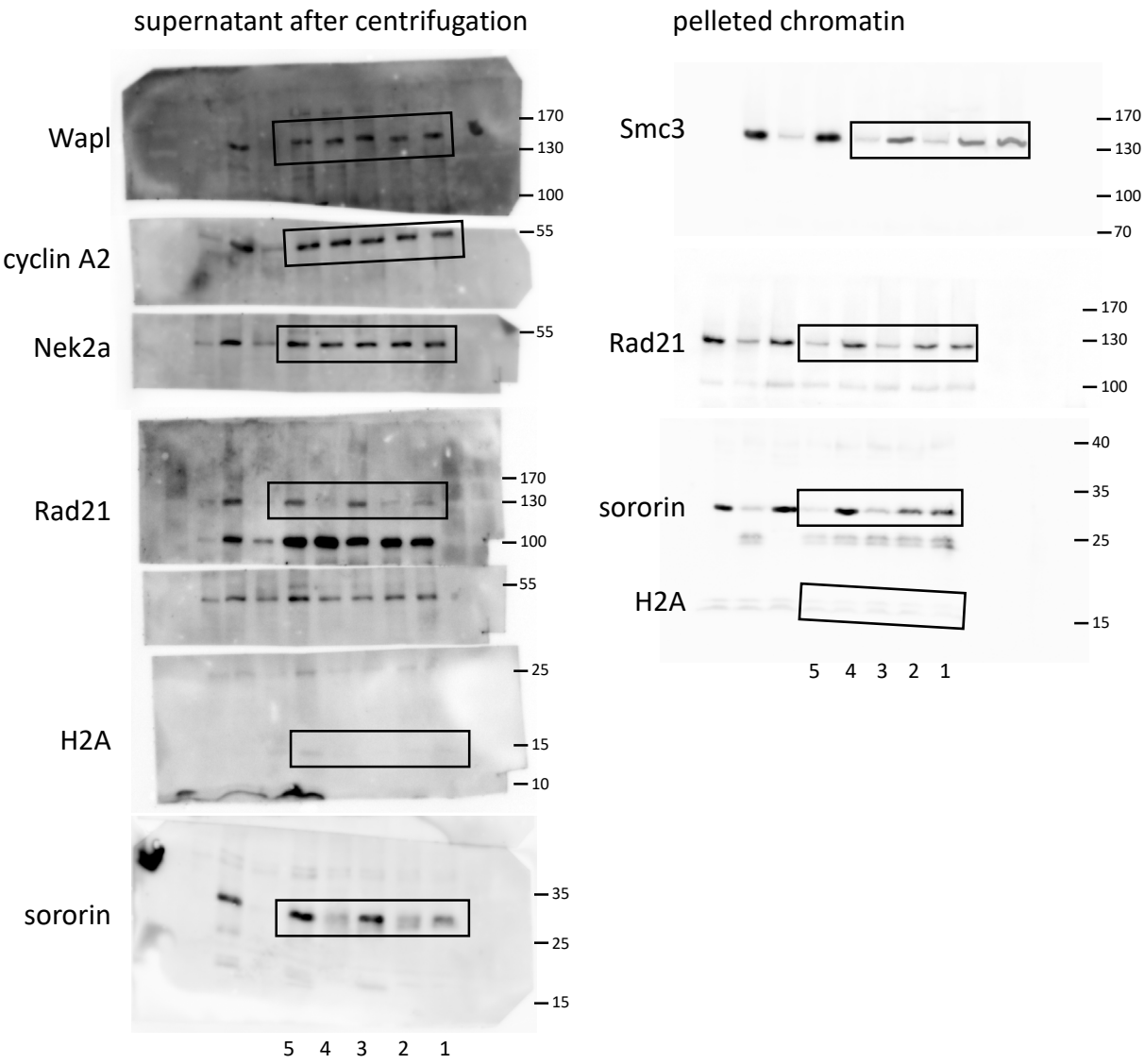

Supplement: Supplementary file 2 — Source data Fig. 1 [file 44318_2024_228_MOESM2_ESM.zip › Figure1/1C.pdf]

Fig.2B

## Chromatin beads

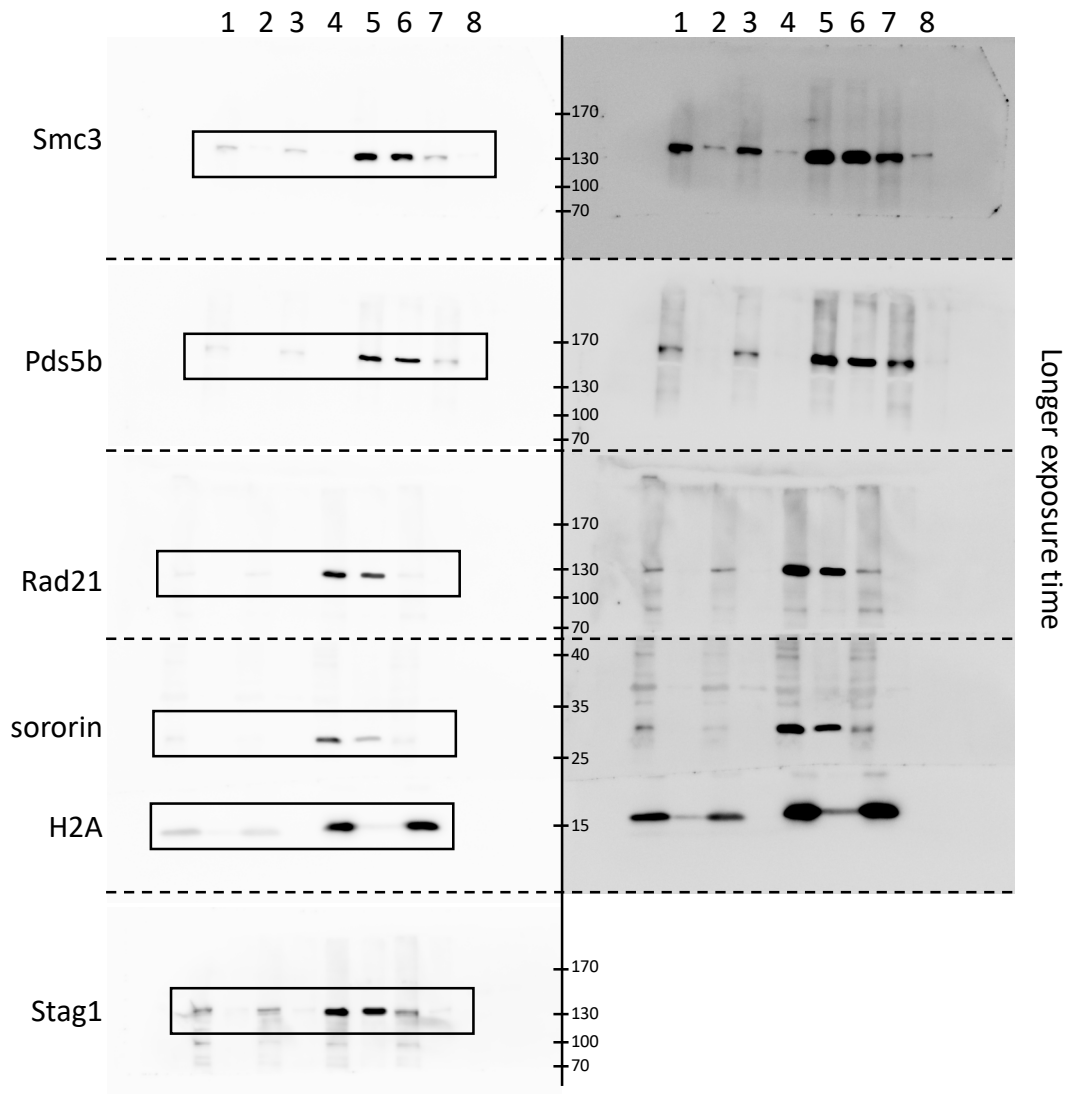

Supplement: Supplementary file 3 — Source data Fig. 2 [file 44318_2024_228_MOESM3_ESM.zip › Figure2/2B.pdf]

Fig.2C

supernatant after centrifugation

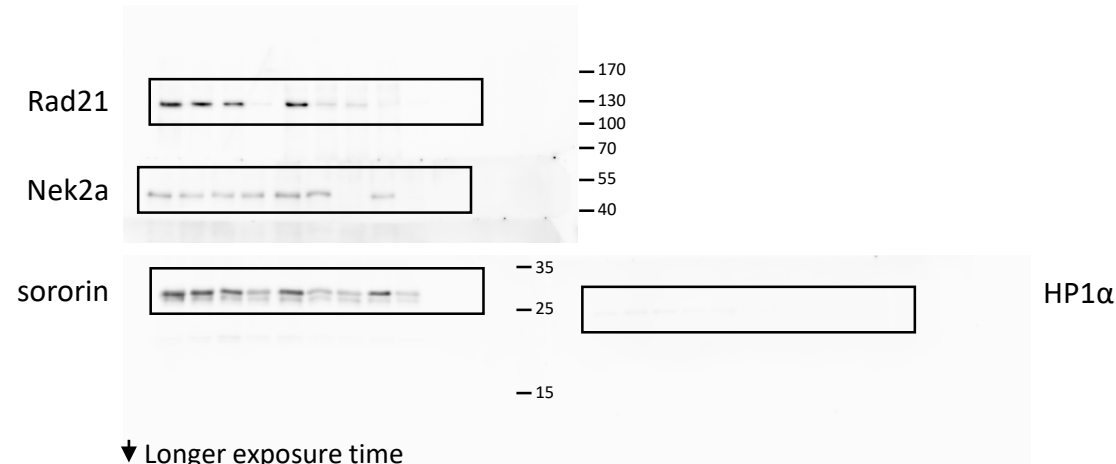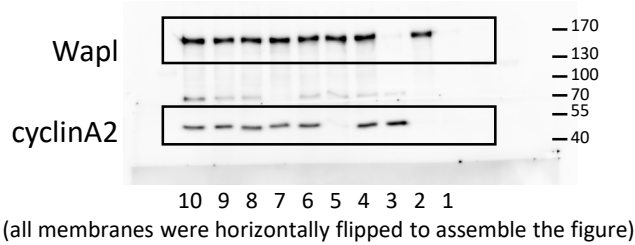

chromatin beads

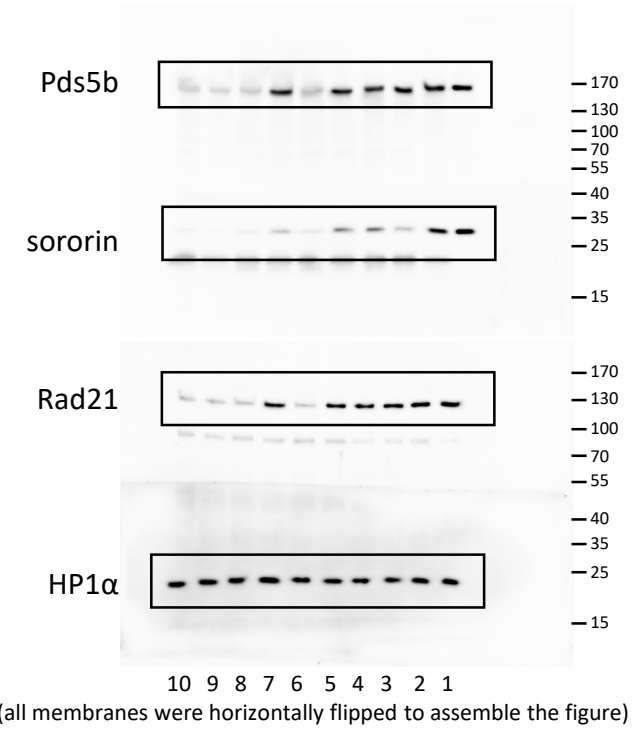

Supplement: Supplementary file 3 — Source data Fig. 2 [file 44318_2024_228_MOESM3_ESM.zip › Figure2/2C.pdf]

Fig.3C

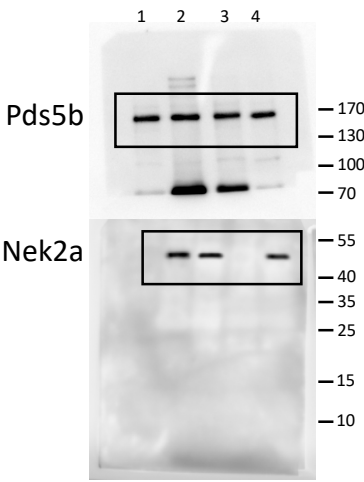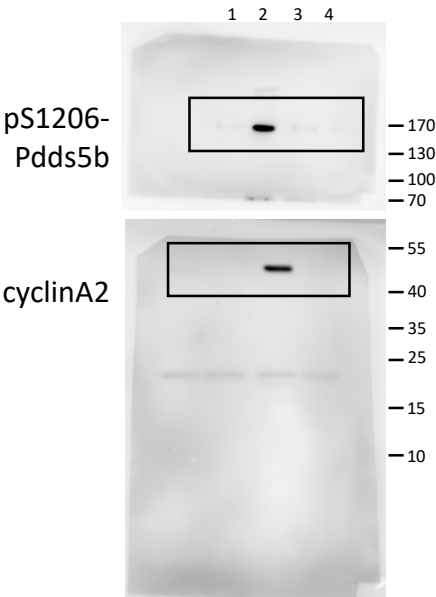

Supplement: Supplementary file 4 — Source data Fig. 3 [file 44318_2024_228_MOESM4_ESM.zip › Figure3/3C.pdf]

Fig. 3C

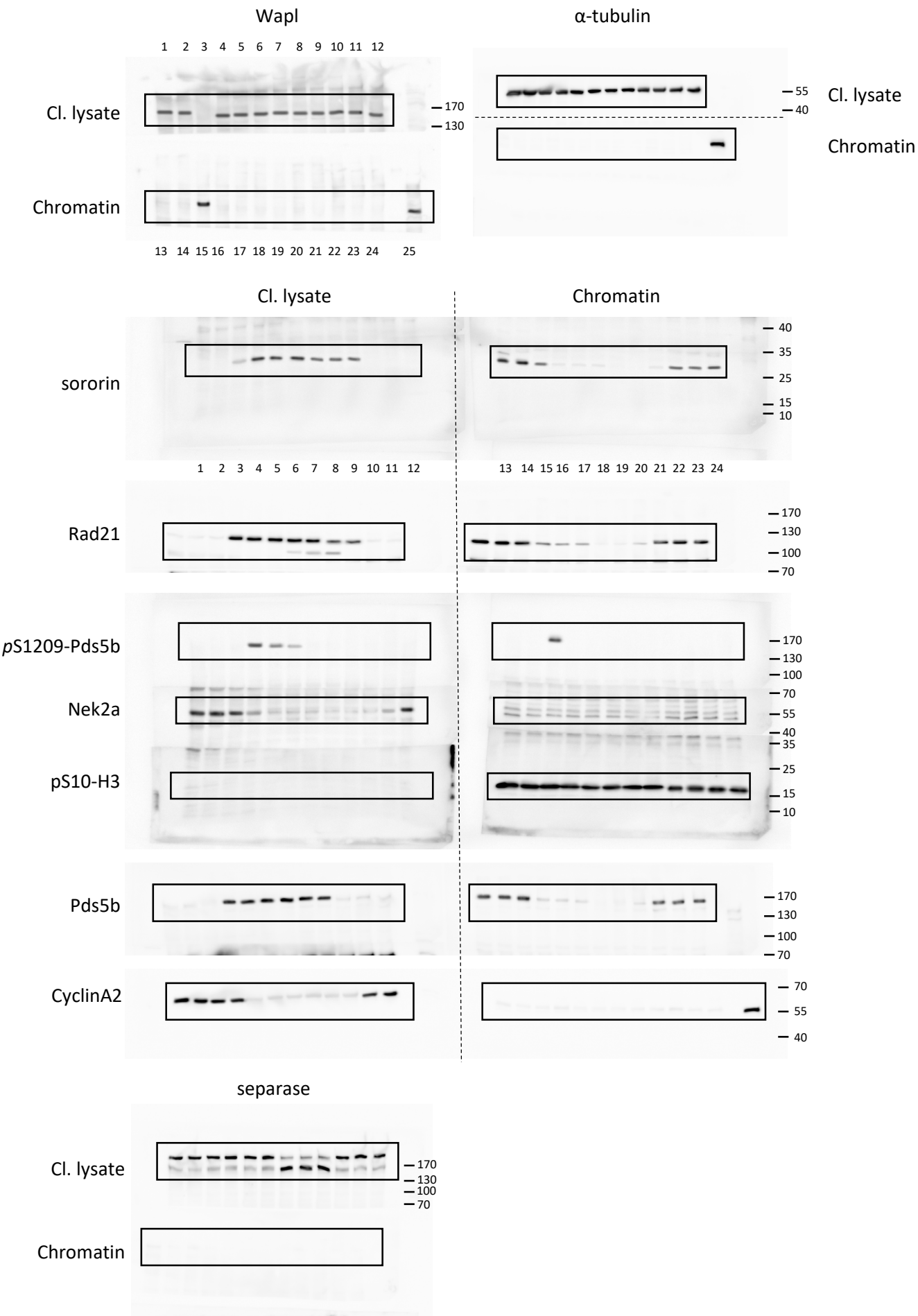

Supplement: Supplementary file 4 — Source data Fig. 3 [file 44318_2024_228_MOESM4_ESM.zip › Figure3/3D.pdf]

Fig.4A

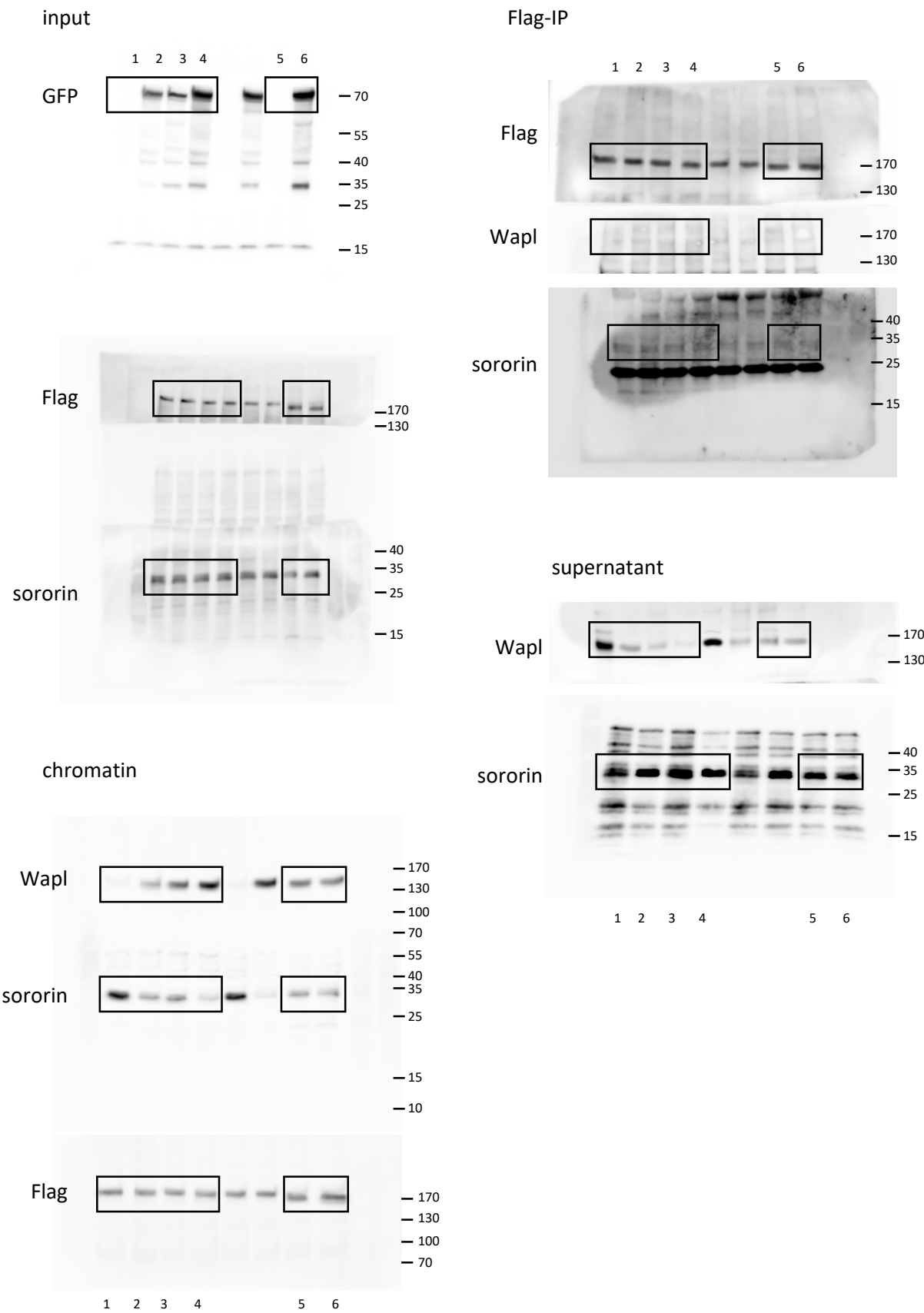

Supplement: Supplementary file 5 — Source data Fig. 4 [file 44318_2024_228_MOESM5_ESM.zip › Figure4/4A.pdf]

Fig.4B

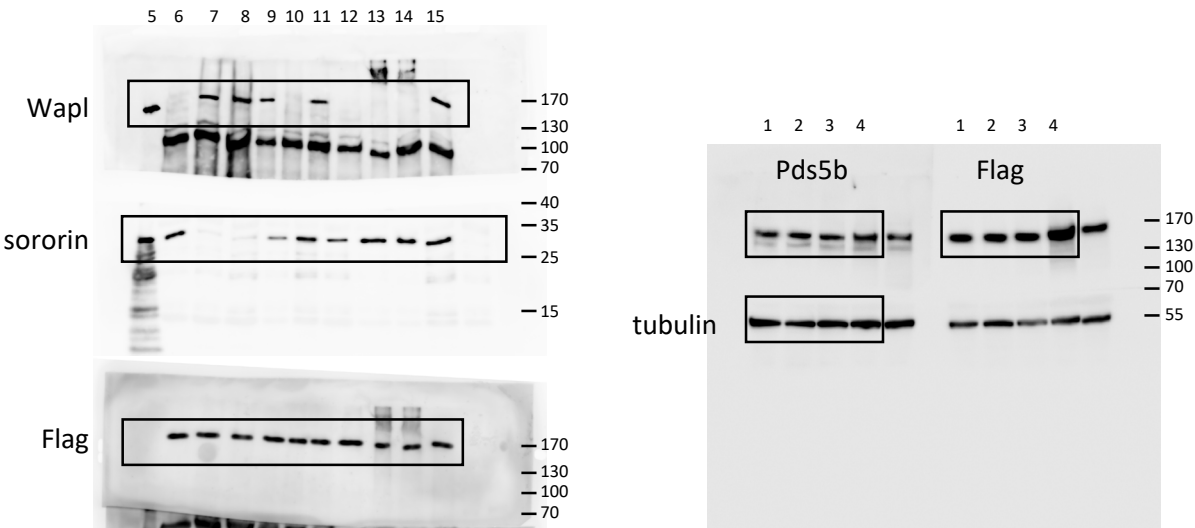

Supplement: Supplementary file 5 — Source data Fig. 4 [file 44318_2024_228_MOESM5_ESM.zip › Figure4/4B.pdf]

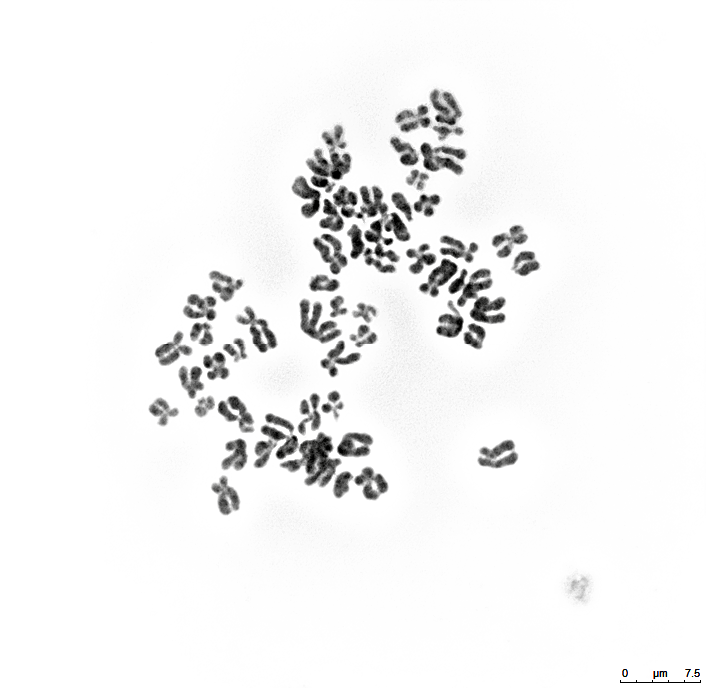

Supplement: Supplementary file 6 — Source data Fig. 5 [file 44318_2024_228_MOESM6_ESM.zip › Figure5/5B_Spreads/butterfly.tif]

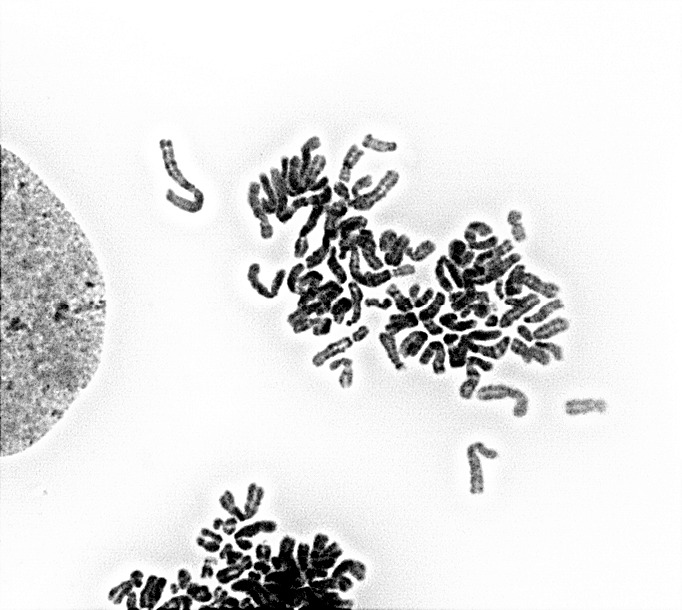

Supplement: Supplementary file 6 — Source data Fig. 5 [file 44318_2024_228_MOESM6_ESM.zip › Figure5/5B_Spreads/long, zipped.tif]

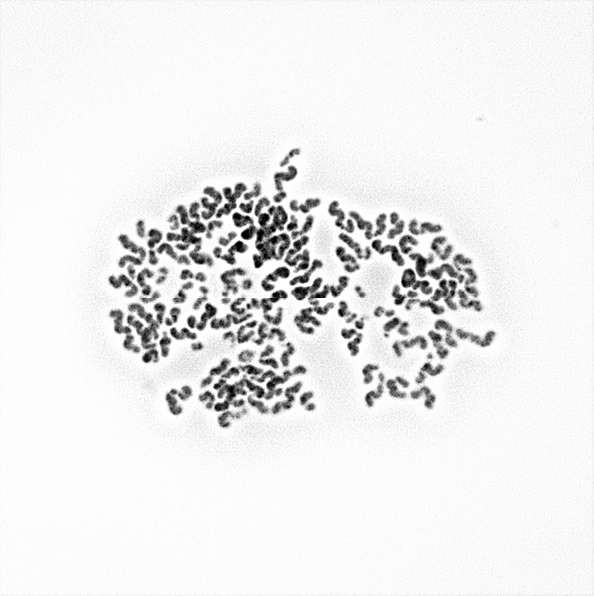

Supplement: Supplementary file 6 — Source data Fig. 5 [file 44318_2024_228_MOESM6_ESM.zip › Figure5/5B_Spreads/separated.tif]

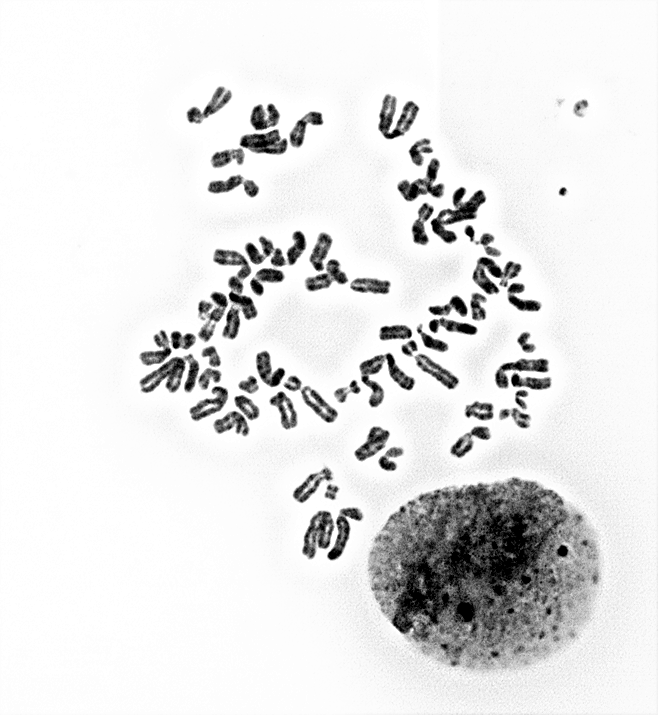

Supplement: Supplementary file 6 — Source data Fig. 5 [file 44318_2024_228_MOESM6_ESM.zip › Figure5/5B_Spreads/zipped.tif]

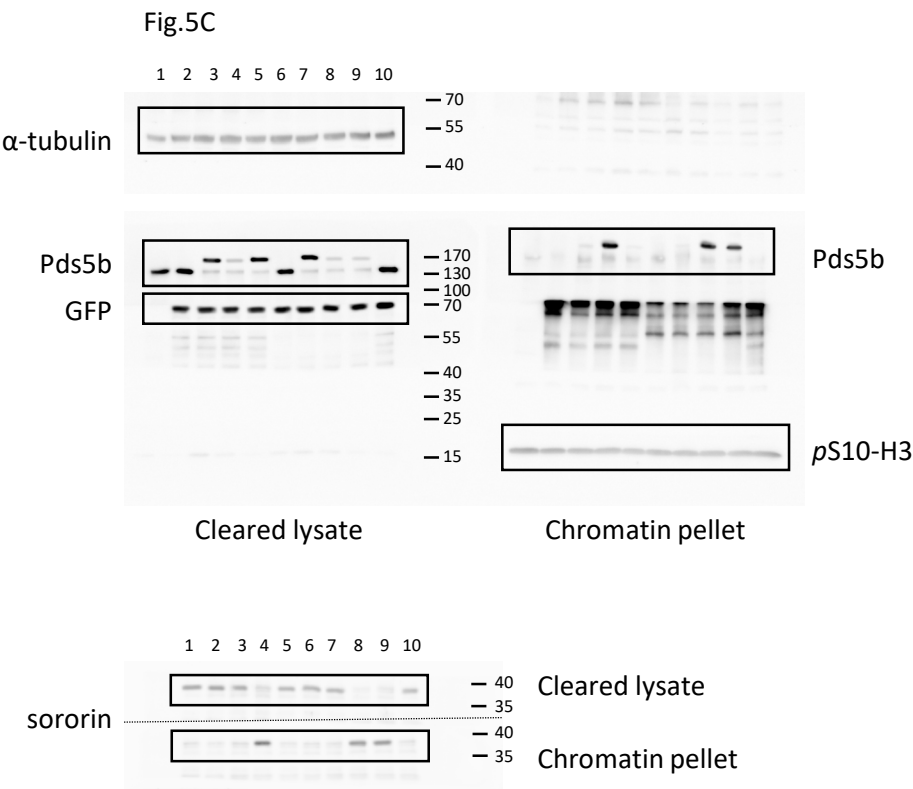

Supplement: Supplementary file 6 — Source data Fig. 5 [file 44318_2024_228_MOESM6_ESM.zip › Figure5/5C.pdf]

Fig.6A

(identical samples were loaded three times in the same direction)

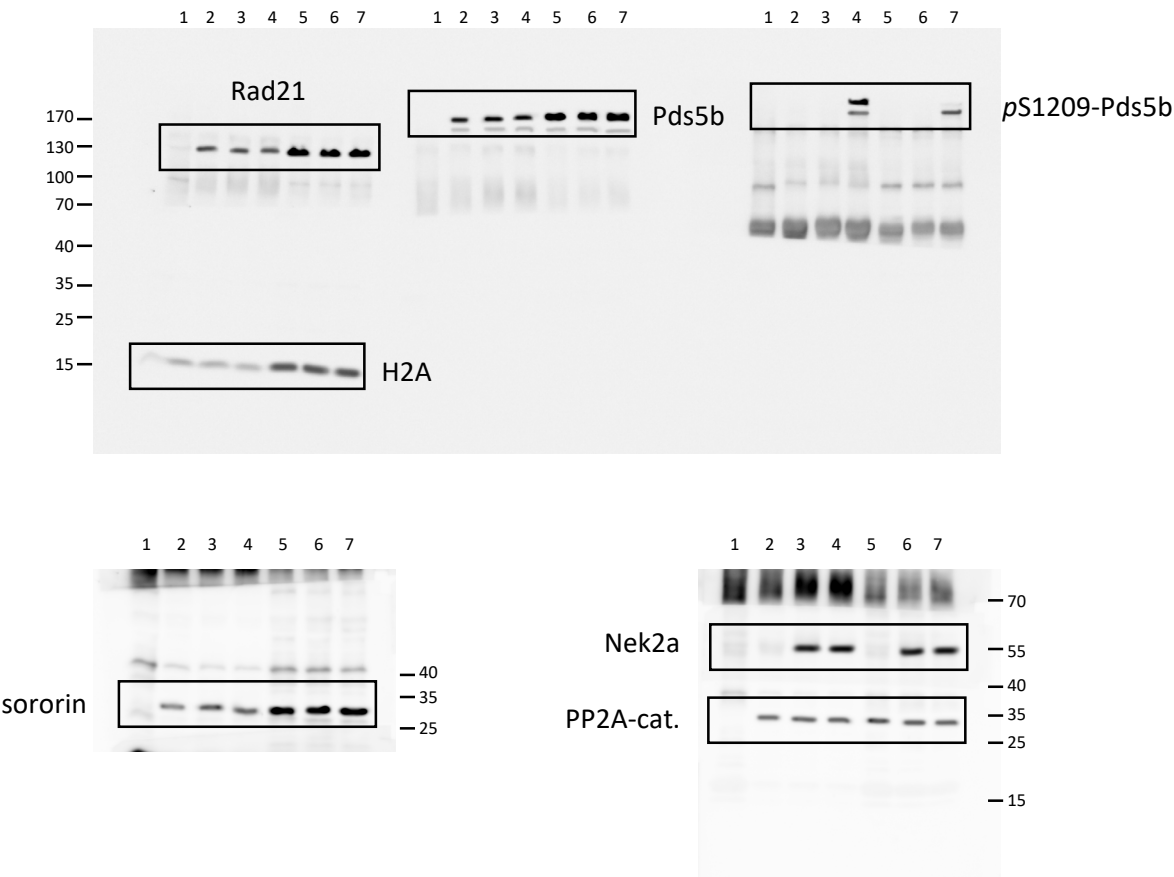

Supplement: Supplementary file 7 — Source data Fig. 6 [file 44318_2024_228_MOESM7_ESM.zip › Figure6/6A.pdf]

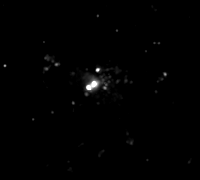

Supplement: Supplementary file 7 — Source data Fig. 6 [file 44318_2024_228_MOESM7_ESM.zip › Figure6/6B/siCONTROL/panPds5b/Crest_pan_GL2.tif]

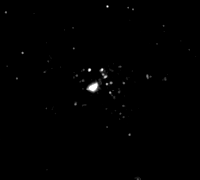

Supplement: Supplementary file 7 — Source data Fig. 6 [file 44318_2024_228_MOESM7_ESM.zip › Figure6/6B/siCONTROL/panPds5b/Pds5B_pan_GL2.tif]

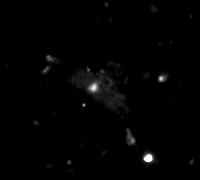

Supplement: Supplementary file 7 — Source data Fig. 6 [file 44318_2024_228_MOESM7_ESM.zip › Figure6/6B/siCONTROL/panPds5b/Rad21_pan_GL2.tif]

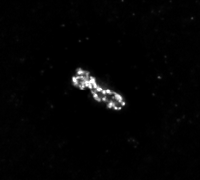

Supplement: Supplementary file 7 — Source data Fig. 6 [file 44318_2024_228_MOESM7_ESM.zip › Figure6/6B/siCONTROL/panPds5b/Smc2_pan_GL2.tif]

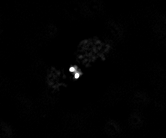

Supplement: Supplementary file 7 — Source data Fig. 6 [file 44318_2024_228_MOESM7_ESM.zip › Figure6/6B/siCONTROL/pS1209Pds5b/Crest_pS1209_GL2.tif]

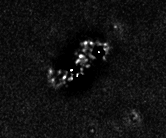

Supplement: Supplementary file 7 — Source data Fig. 6 [file 44318_2024_228_MOESM7_ESM.zip › Figure6/6B/siCONTROL/pS1209Pds5b/Pds5B_pS1209_GL2.tif]

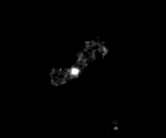

Supplement: Supplementary file 7 — Source data Fig. 6 [file 44318_2024_228_MOESM7_ESM.zip › Figure6/6B/siCONTROL/pS1209Pds5b/Rad21_pS1209_GL2.tif]

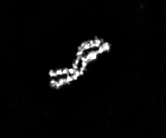

Supplement: Supplementary file 7 — Source data Fig. 6 [file 44318_2024_228_MOESM7_ESM.zip › Figure6/6B/siCONTROL/pS1209Pds5b/Smc2_pS1209_GL2.tif]

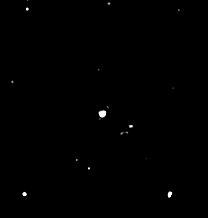

Supplement: Supplementary file 7 — Source data Fig. 6 [file 44318_2024_228_MOESM7_ESM.zip › Figure6/6B/siWAPL/panPds5b/Crest_pan_siWapl.tif]

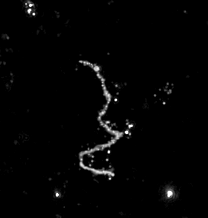

Supplement: Supplementary file 7 — Source data Fig. 6 [file 44318_2024_228_MOESM7_ESM.zip › Figure6/6B/siWAPL/panPds5b/Pds_pan_siWapl.tif]

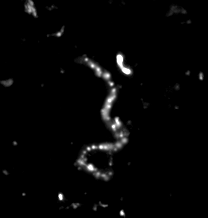

Supplement: Supplementary file 7 — Source data Fig. 6 [file 44318_2024_228_MOESM7_ESM.zip › Figure6/6B/siWAPL/panPds5b/Rad21_pan_siWapl.tif]

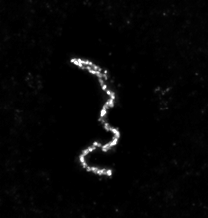

Supplement: Supplementary file 7 — Source data Fig. 6 [file 44318_2024_228_MOESM7_ESM.zip › Figure6/6B/siWAPL/panPds5b/Smc2_pan_siWapl.tif]

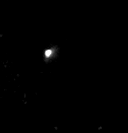

Supplement: Supplementary file 7 — Source data Fig. 6 [file 44318_2024_228_MOESM7_ESM.zip › Figure6/6B/siWAPL/pS1209Pds5b/Crest_pS1209_siWapl.tif]

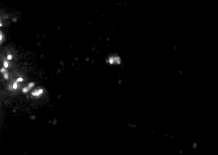

Supplement: Supplementary file 7 — Source data Fig. 6 [file 44318_2024_228_MOESM7_ESM.zip › Figure6/6B/siWAPL/pS1209Pds5b/CREST_siWAPL.tif]

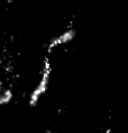

Supplement: Supplementary file 7 — Source data Fig. 6 [file 44318_2024_228_MOESM7_ESM.zip › Figure6/6B/siWAPL/pS1209Pds5b/Pds5B_pS1209_siWapl.tif]

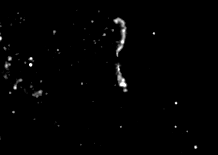

Supplement: Supplementary file 7 — Source data Fig. 6 [file 44318_2024_228_MOESM7_ESM.zip › Figure6/6B/siWAPL/pS1209Pds5b/pS1209Pds5B_siWAPL.tif]

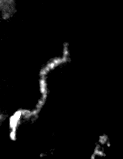

Supplement: Supplementary file 7 — Source data Fig. 6 [file 44318_2024_228_MOESM7_ESM.zip › Figure6/6B/siWAPL/pS1209Pds5b/Rad21_pS1209_siWapl.tif]

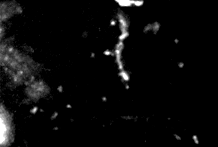

Supplement: Supplementary file 7 — Source data Fig. 6 [file 44318_2024_228_MOESM7_ESM.zip › Figure6/6B/siWAPL/pS1209Pds5b/Rad21_siWAPL.tif]

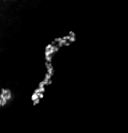

Supplement: Supplementary file 7 — Source data Fig. 6 [file 44318_2024_228_MOESM7_ESM.zip › Figure6/6B/siWAPL/pS1209Pds5b/Smc2_pS1209_siWapl.tif]

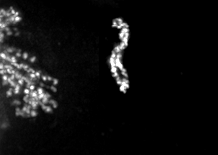

Supplement: Supplementary file 7 — Source data Fig. 6 [file 44318_2024_228_MOESM7_ESM.zip › Figure6/6B/siWAPL/pS1209Pds5b/Smc2_siWAPL.tif]

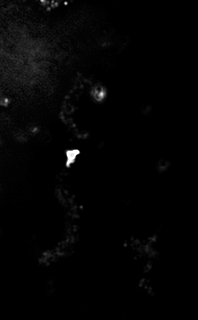

Supplement: Supplementary file 7 — Source data Fig. 6 [file 44318_2024_228_MOESM7_ESM.zip › Figure6/6B/siWAPL_siPDS5B/panPds5b/Crest_siWAPL_PDS5B.tif]

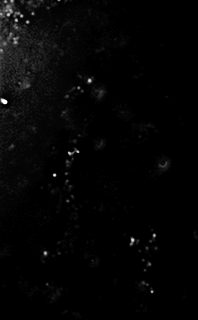

Supplement: Supplementary file 7 — Source data Fig. 6 [file 44318_2024_228_MOESM7_ESM.zip › Figure6/6B/siWAPL_siPDS5B/panPds5b/panPds5B_siWAPL_PDS5B.tif]

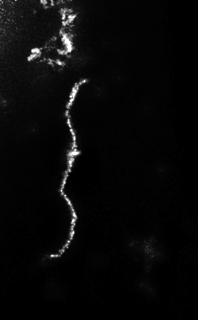

Supplement: Supplementary file 7 — Source data Fig. 6 [file 44318_2024_228_MOESM7_ESM.zip › Figure6/6B/siWAPL_siPDS5B/panPds5b/Rad21_siWAPL_PDS5B.tif]

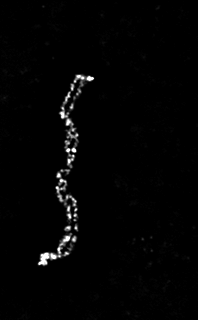

Supplement: Supplementary file 7 — Source data Fig. 6 [file 44318_2024_228_MOESM7_ESM.zip › Figure6/6B/siWAPL_siPDS5B/panPds5b/Smc2_siWAPL_PDS5B.tif]

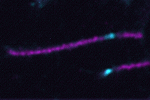

Supplement: Supplementary file 7 — Source data Fig. 6 [file 44318_2024_228_MOESM7_ESM.zip › Figure6/6B/siWAPL_siSGO1/6B_siWapl_pS1209.tif]

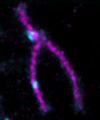

Supplement: Supplementary file 7 — Source data Fig. 6 [file 44318_2024_228_MOESM7_ESM.zip › Figure6/6B/siWAPL_siSGO1/6B_siWapl_siSgo1_pS1209.tif]

Fig. 7A

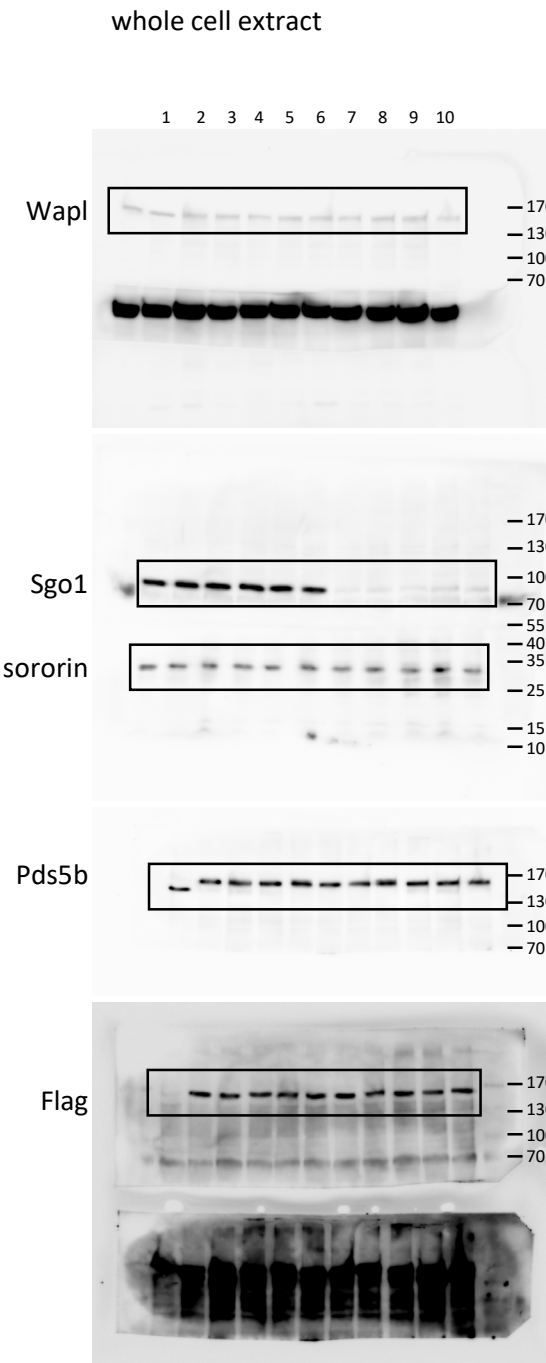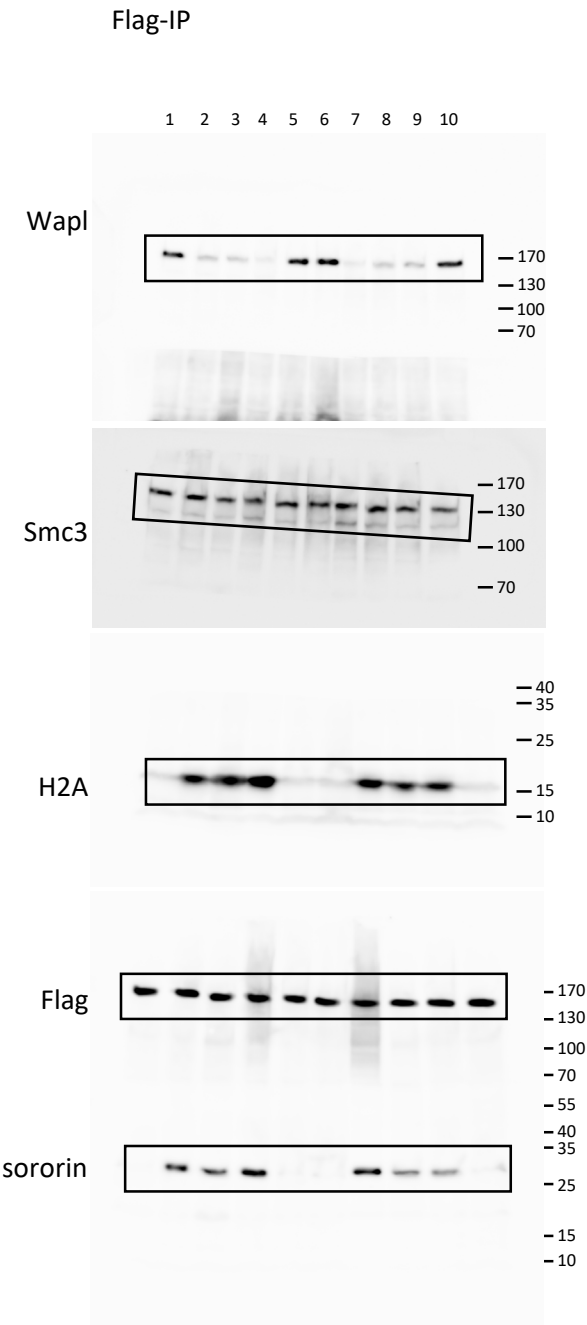

Supplement: Supplementary file 8 — Source data Fig. 7 [file 44318_2024_228_MOESM8_ESM.zip › Figure7/7A.pdf]

Fig. 8B

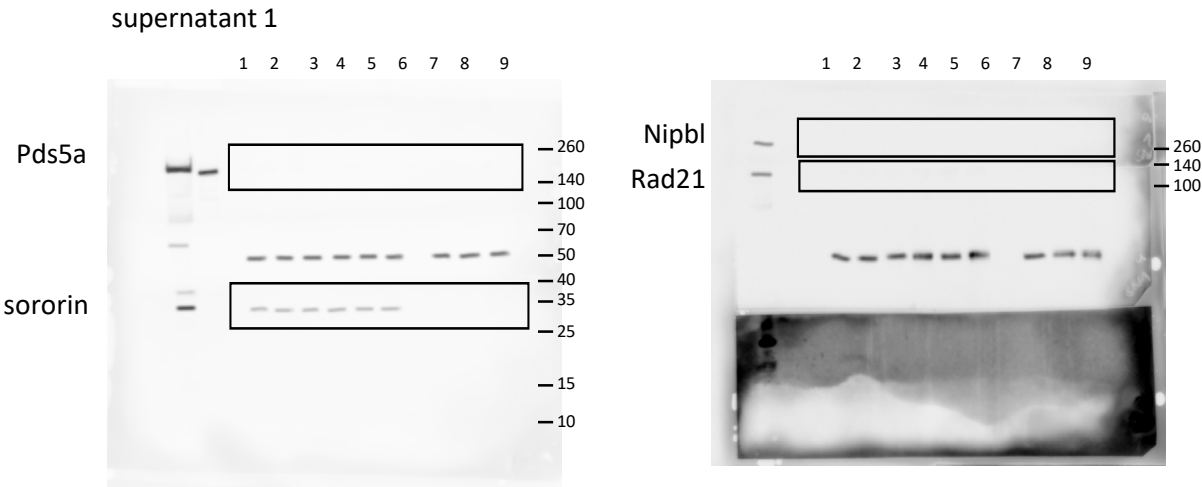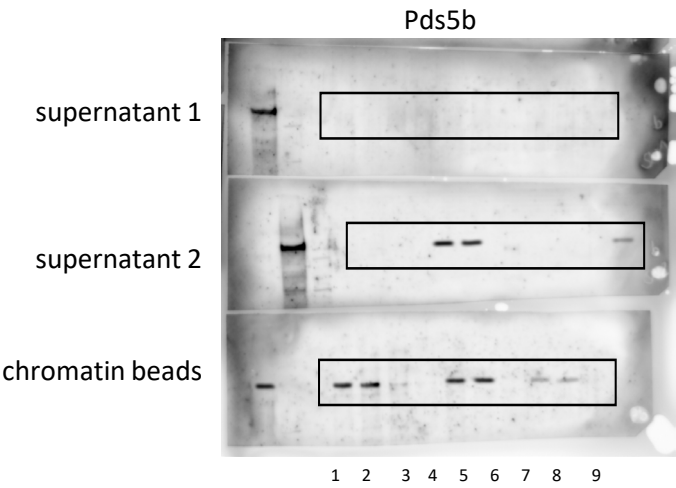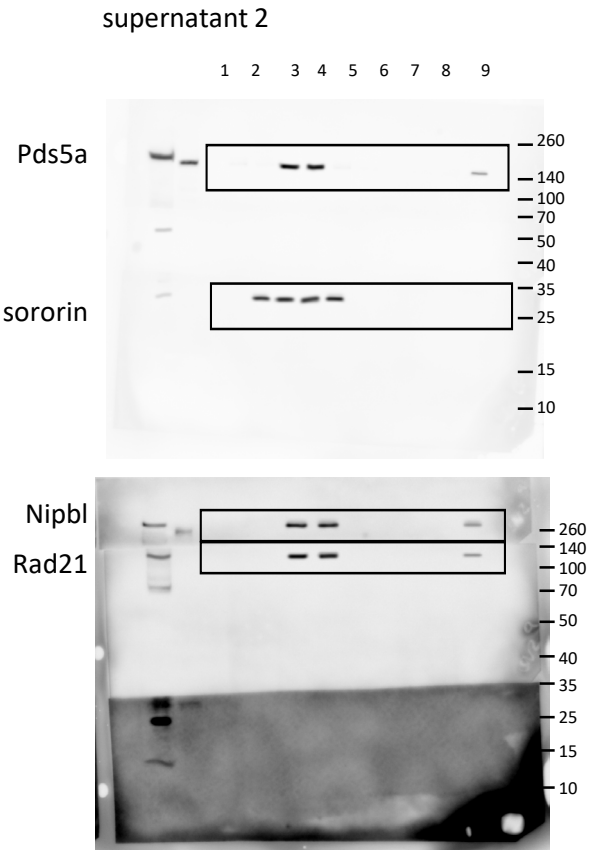

Fig. 8B

Chromatin beads

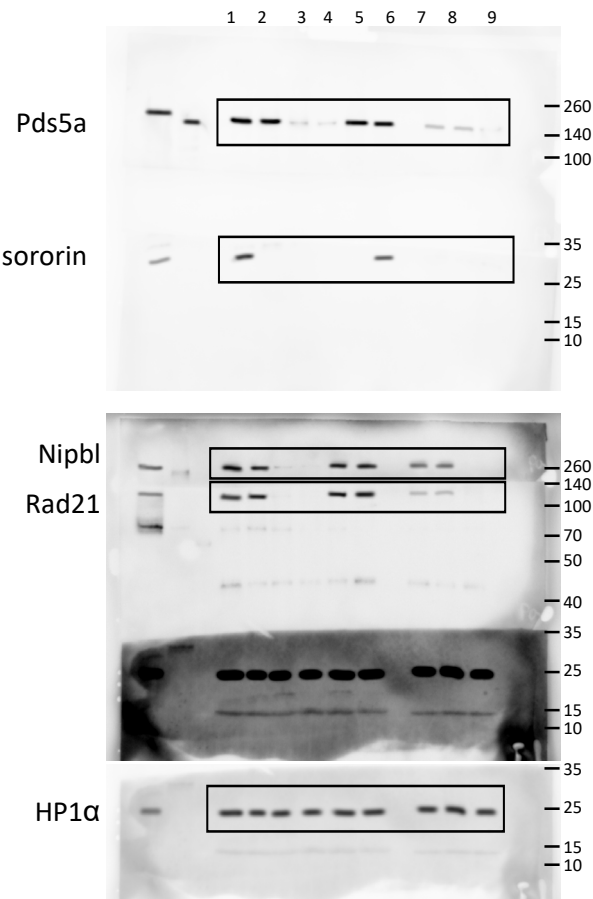

(shorter exposure)

Supplement: Supplementary file 9 — Source data Fig. 8 [file 44318_2024_228_MOESM9_ESM.zip › Figure8/8B.pdf]
