# Supplementary material for: Requirement of Nek2a and cyclin A2 for Wapl-dependent removal of cohesin from prophase chromatin
Source: EMBO J. 2024 Sep 13;43(21):20. doi: 10.1038/s44318-024-00228-9 (PMC11535040; doi:10.1038/s44318-024-00228-9)

Source Data: EMBOJ-2024-117218

Fig.3A

Autoradiography

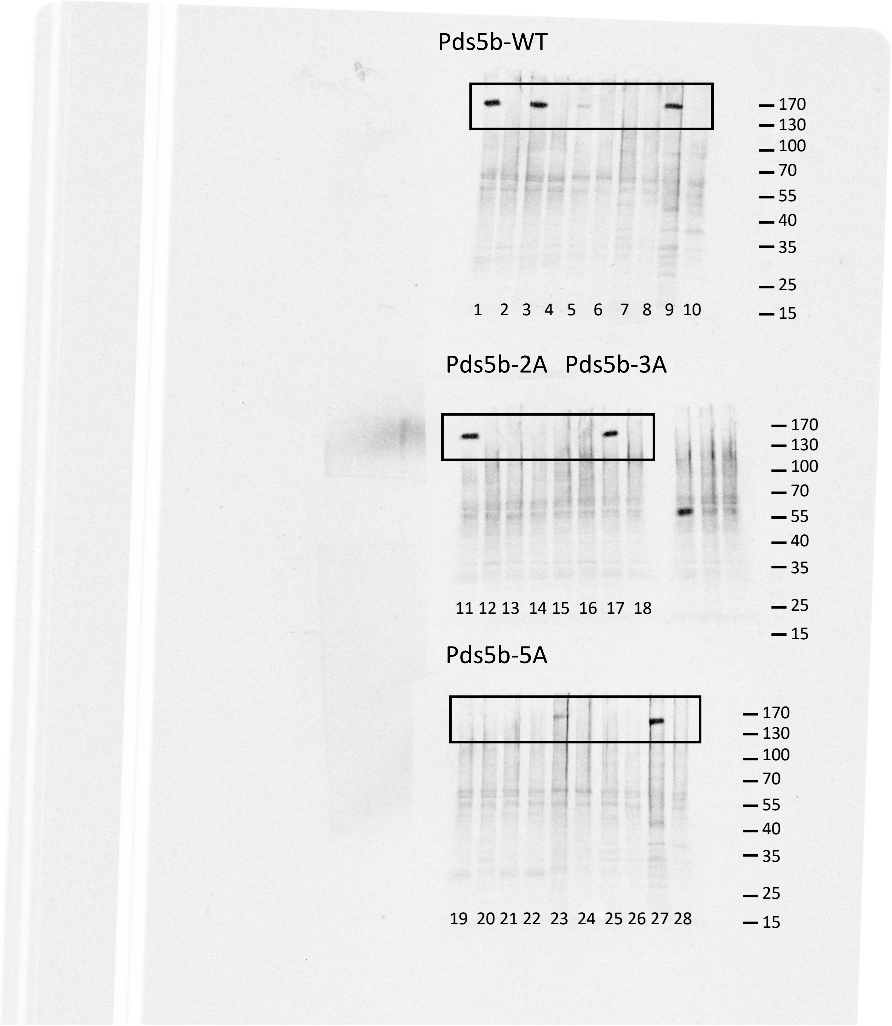

Immunoblot (identical PVDF-membrane used for autoradiography was probed with Pds5b antibody)

Pds5b-WT

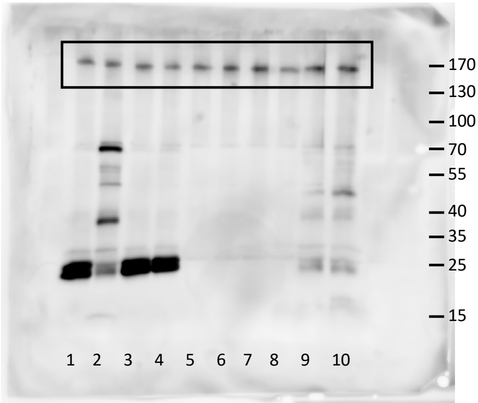

Pds5b-2A Pds5b-3A

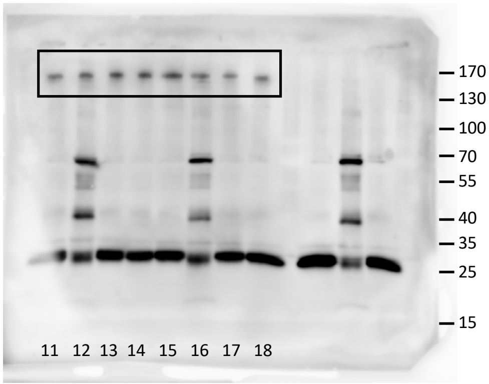

Pds5b-5A

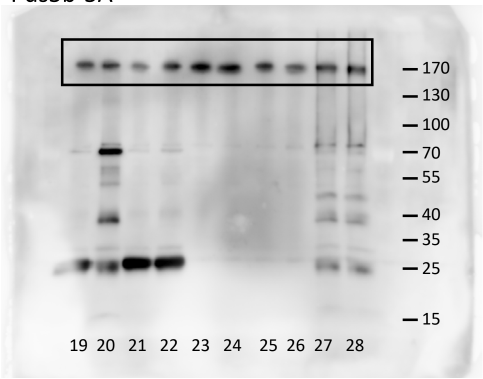

Supplement: Supplementary file 4 — Source data Fig. 3 [file 44318_2024_228_MOESM4_ESM.zip › Figure3/3A.pdf]
