# Supplementary material for: Requirement of Nek2a and cyclin A2 for Wapl-dependent removal of cohesin from prophase chromatin
Source: EMBO J. 2024 Sep 13;43(21):20. doi: 10.1038/s44318-024-00228-9 (PMC11535040; doi:10.1038/s44318-024-00228-9)
Supplement: Supplementary file 10 — Expanded View Figures [file 44318_2024_228_MOESM10_ESM.pdf]

Expanded View Figures

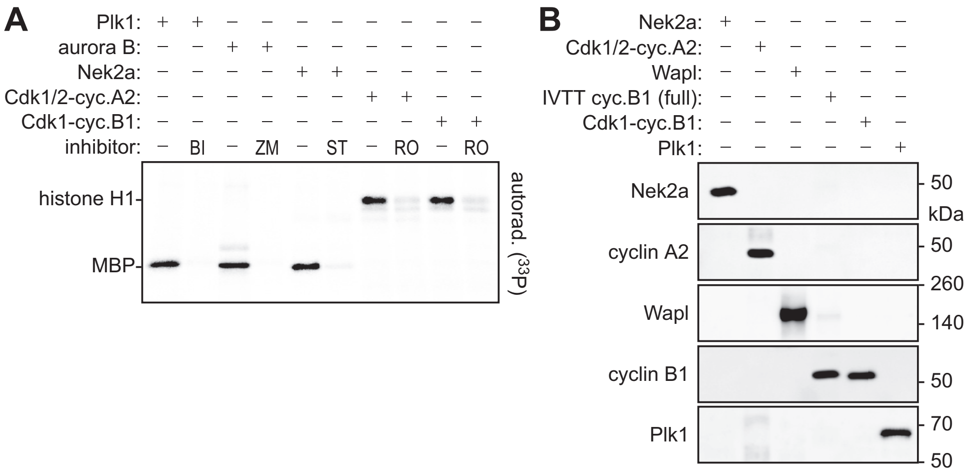

Figure EV1. Characterization of recombinant kinases.

(**A**) Recombinant kinases are active as judged by the phosphorylation of model substrates. Plk1, aurora B-INCENP, Nek2a, Cdk1/2-cyclin A2, or Cdk1-cyclin B1 supplemented with their specific inhibitor or carrier solvent DMSO (−) were incubated with the corresponding model substrate in the presence of [γ^{33}P]-ATP. Reactions were subjected to SDS-PAGE followed by autoradiography. BI BI2536, ZM ZM-447439, ST staurosporine, RO RO-3306, MBP myelin basic protein. (**B**) Recombinant Nek2a, Cdk1/2-cyclin A2, and Wapl are free of cyclin B1 and Plk1. The preparations of Nek2a, Cdk1/2-cyclin A2, Wapl, Cdk1-cyclin B1, and Plk1 used for the release assays were characterized by immunoblotting using the indicated antibodies. In vitro expressed (IVTT) cyclin B1 served as an additional control.

 © The Author(s)

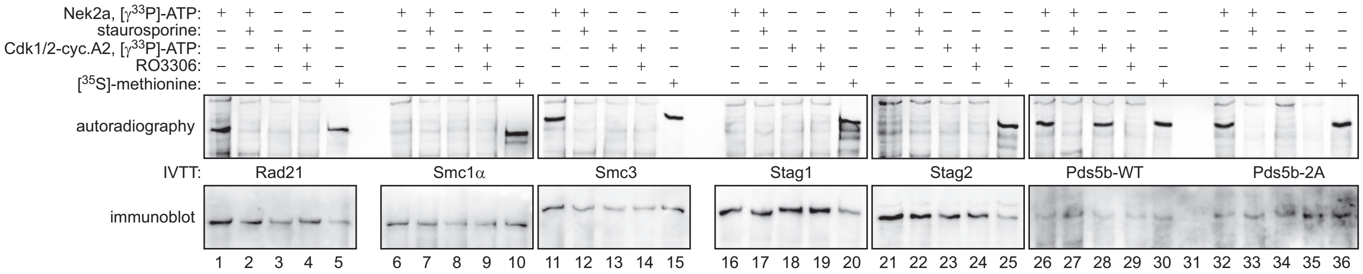

Figure EV2. Nek2a and Cdk1/2-cyclin A2 both phosphorylate in vitro expressed Pds5b.

Cohesin subunits and Pds5b variants were expressed by coupled in vitro transcription-translation (IVTT), incubated with Nek2a, Cdk1/2-cyclin A2, [γ-^{33}P]-ATP, and kinase inhibitors, as indicated, separated by SDS-PAGE and analyzed by autoradiography and immunoblotting. ^{35}S-methionine labeled IVTT products served as indicators of the migration behavior of the respective full-length protein. Note that Pds5b-2A (Ser1161,1166Ala) is resistant to phosphorylation by Cdk1/2-cyclin A2 but not Nek2a.

© The Author(s)

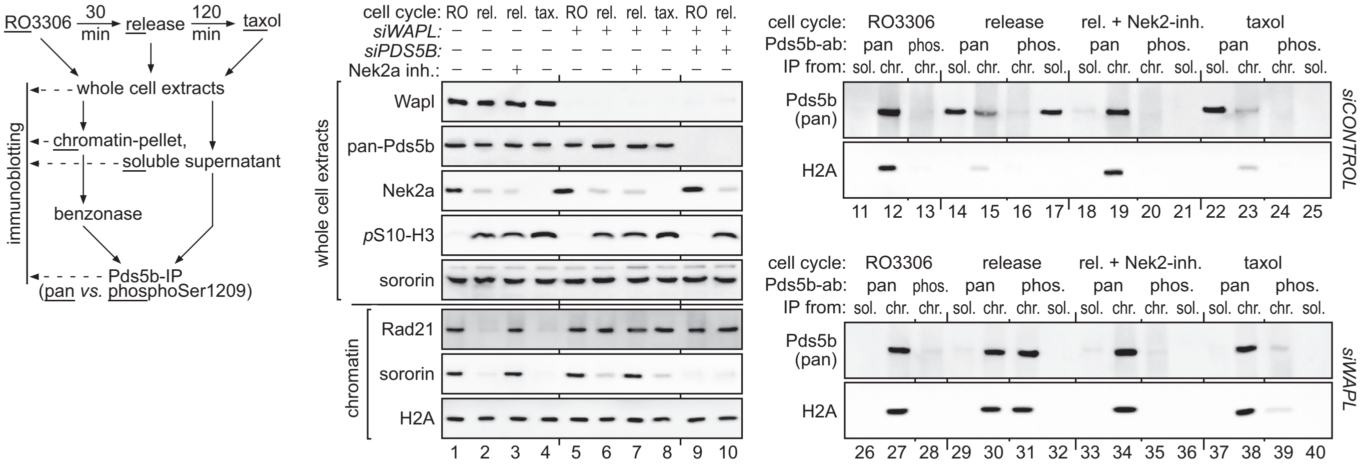

Figure EV3. Pds5b phosphorylated on Ser1209 by Nek2a exhibits Wapl-dependent displacement from early mitotic chromatin.

HeLaK cells transfected to deplete Pds5b and/or Wapl by RNAi were synchronized and harvested in G2-, pro-, and prometaphase, fractionated into chromatin and soluble lysate and subjected to the indicated (IP-) Western analyses. Nek2a inhibitor (NCL 00017509) was added at the time of release from RO-3306 and cells were harvested 30 min thereafter.

 © The Author(s)

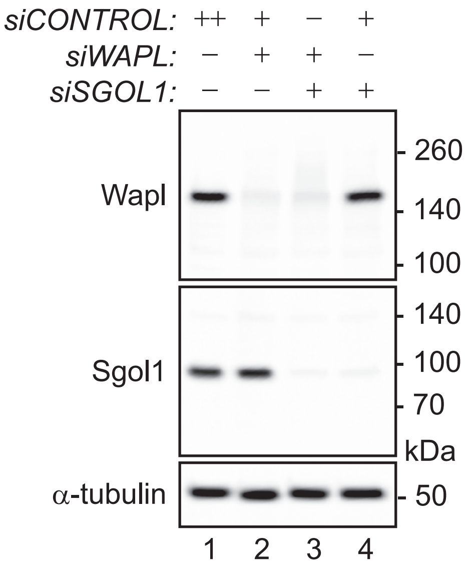

Figure EV4. Wapl and/or Sgo1 are efficiently depleted by RNAi.

HeLaK cells transfected with the indicated siRNAs during a thymidine arrest were released into a G2- and, from there into a prometaphase arrest prior to their analysis by immunoblotting. Aliquots of the cells analyzed in lanes 2 and 3 were subjected to spread-IFs as shown in Fig. 6B (lower row panels).
